# PDM-ProValue meets cardiovascular outcome trials in diabetes

**DOI:** 10.1186/s12933-019-0815-3

**Published:** 2019-01-28

**Authors:** Katharina Fritzen, Oliver Schnell

**Affiliations:** 1 Sciarc GmbH, Baierbrunn, Germany; 2 Forschergruppe Diabetes e.V., Muenchen-Neuherberg, Germany

**Keywords:** PDM-ProValue, iPDM, CVOTs, Diabetes, SMBG

## Abstract

Novel studies have increased our knowledge regarding optimal treatment options in diabetes. Key studies that have broadened our knowledge about optimal treatment options in diabetes in recent years are cardiovascular outcome trials (CVOTs) and studies investigating aspects of digitalisation and monitoring of glucose (e.g. PDM-ProValue). We aimed at highlighting similarities between the two important pillars for a successful diabetes management. We emphasise the need for a consideration of both approaches in future clinical trial designs and protocols.

## Introduction

Novel studies have increased our knowledge regarding optimal treatment options in diabetes. A key pillar are cardiovascular (CV) outcome trials (CVOTs) that focus primarily on safety of glucose-lowering medication. Multiple CVOTs have been published on the safety of sodium-glucose cotransporter-2 (SGLT-2) inhibitors, glucagon-like peptide-1 (GLP-1) receptor agonists, dipeptidyl peptidase-4 (DPP-4) inhibitors and selected insulins. A further important pillar of studies includes aspects of digitalisation and monitoring of glucose. The recently published PDM-ProValue Study can be seen as an example of a study focusing on effects of modern digitalisation and integrated personalised diabetes management in patients with type 2 diabetes that are treated with insulin. Successful diabetes management requires both novel treatment strategies and innovative glucose monitoring. Both aspects are of great importance and results of both study areas should be considered, when it comes to an incorporation of results into clinical diabetes care, individual treatment decisions and into guidelines on diabetes treatment and management.

Although both study approaches have different aims and structures it is of high clinical interest to compare both elements. Thus, the aim of this report was to highlight similarities of the two important pillars of recent studies, new diabetes technologies in glucose monitoring and digitalisation (e.g. PDM-ProValue study) and safety of treatment options (e.g. CVOTs), to emphasise the need for a hand-in-hand progression of clinical trial design and protocols.

### iPDM—PDM-ProValue: Study design and outcomes

**Fig. 1:**
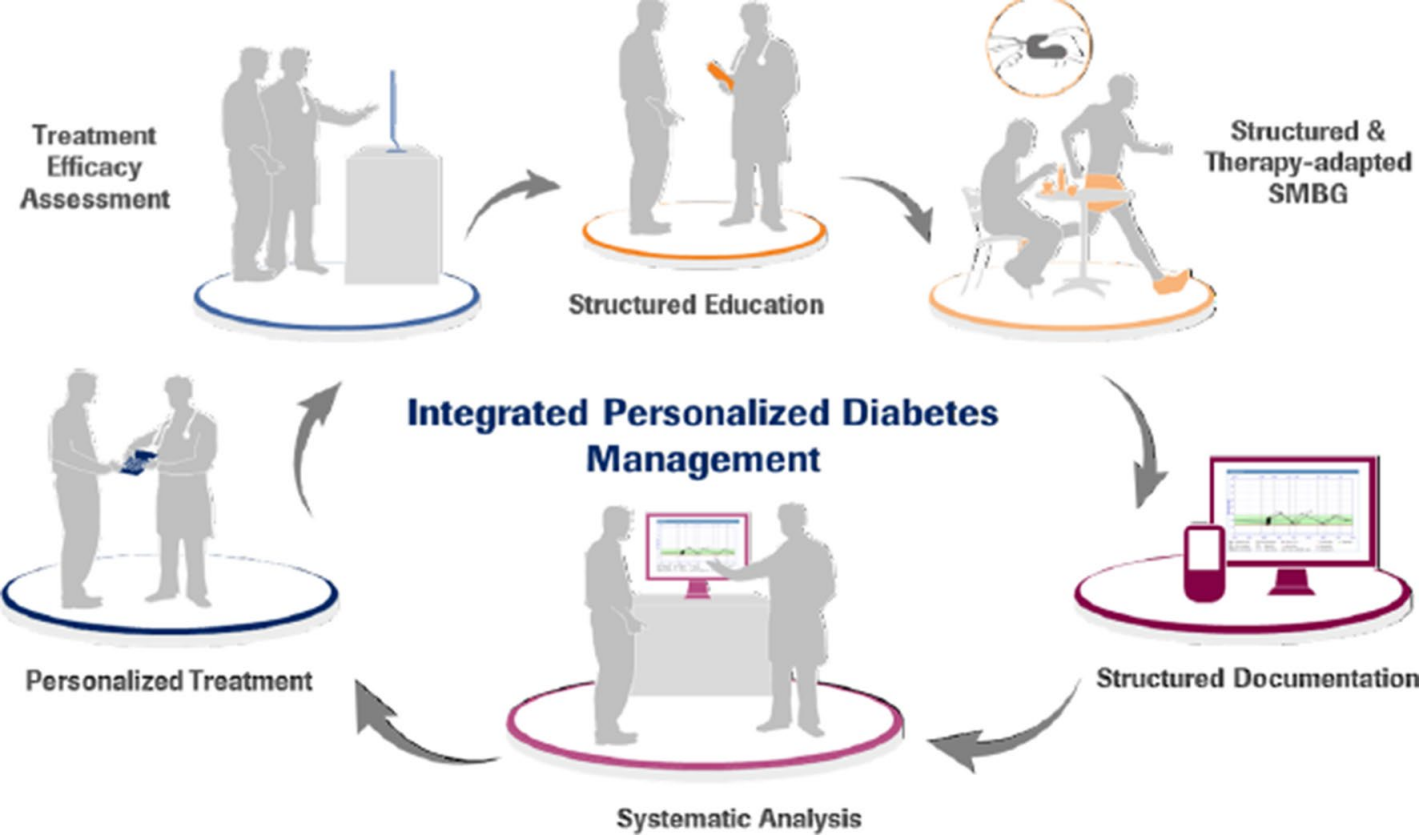
The iPDM process. iPDM is defined as an interactive, six-step structured intervention process containing the following components: (1) structured education, (2) structured and therapy-adapted self-monitoring of blood glucose, (3) structured documentation, (4) systematic analysis, (5) personalised treatment and (6) treatment effectiveness assessment [2]

The PDM-ProValue study investigated the influence of treatment according to integrated personalised diabetes management (iPDM) in comparison with standard treatment in patients with insulin-treated type 2 diabetes (T2DM). In the study, iPDM is defined as an interactive, six-step structured intervention process containing the following components: (1) structured assessment and patient education, (2) structured and therapy-adapted self-monitoring of blood glucose (SMBG), (3) structured documentation, (4) systematic analysis, (5) personalised treatment and (6) treatment efficacy assessment (Fig. 1). The recurring sequences aim at improving glycaemic control of patients with diabetes by supporting the doctor-patient-relationship and therapeutic decisions with a structured process and digital technology.

The endpoints of the PDM-ProValue study were improvement in glycaemic control, assessed by changes in HbA1c, percentage of patients achieving > 0.5% (> 6 mmol/mol) HbA1c reduction, diabetes therapy adjustments, change in SMBG testing frequency, various patient-related outcomes (PROs) as well as physician-related outcomes. The study was conducted as a 12-month, prospective, controlled, cluster-randomised study. 101 German medical practices (general practitioners and specialised diabetes practices) were enrolled, 53 randomised in the iPDM and 48 in the control group (907 participants, 440 in the intervention and 467 in the control group). The control group was treated with usual care whereas patients in the iPDM group followed the six steps that are included in the digitalised structured intervention process. At 12 months, glycaemic control improved in both groups compared to baseline, with a between-group difference of 0.2% (p < 0.0324). The incidence of hypoglycaemic episodes (defined as blood glucose (BG) level < 70 mg/dl) was similar in both treatment groups. A higher percentage of patients in the iPDM group had a change in insulin therapy during their study participation. Overall, the results of the study program showed that iPDM can improve not only glycaemic control by reducing HbA1c values but also treatment adjustments, adherence to treatment regimen and patient and physician satisfaction [1, 2]. Thus the PDM-ProValue study confirms positive results of earlier trials, showing that structured SMBG monitoring and the appropriate use of digitalised information management systems significantly improves glycaemic control and facilitates timely treatment changes [3–5].

### CVOTs in diabetes

Since 2008, when the Food and Drug Administration issued a guidance for the approval of glucose-lowering medication with regard to CV safety, twelve long-term prospective CVOTs have been completed, whilst 10 further studies are currently running. The completed and published studies investigated CV safety of four DPP-4 inhibitors (saxagliptin, alogliptin, sitagliptin and linagliptin [6–9]), five GLP-1 receptor agonists (lixisenatide, liraglutide, semaglutide, exenatide and albiglutide [10–14]) and three SGLT-2 inhibitors (empagliflozin, canagliflozin and dapagliflozin [15–17]). Also Insulin glargine and Insulin degludec have been subject of CVOTs [18–20]. Collectively, non-inferiority of these drugs to placebo with regard to the major adverse cardiac event (MACE) primary composite end point could be confirmed. In addition to non-inferiority five trials (LEADER, SUSTAIN-6, Harmony Outcomes, EMPA-REG OUTCOME and CANVAS) could show the capability of the investigated component (liraglutide, semaglutide, albiglutide, empagliflozin and canagliflozin, respectively) to reduce CV outcomes in diabetes patients with high CV risk. Glycaemic control and HbA1c were determined but not the focus of these studies. Currently published CVOTs include participants with diabetes and either established CV disease (CVD) or at high risk of CVD, thus not being adequately representative of the general population [21].

### PDM-ProValue and CVOTs: Comparison of patient characteristics

The characteristics of participants of PDM-ProValue and CVOTs are summarised in Tables 1 and 2. The populations of the two study approaches have a number of similarities: T2DM patients, more male than female participants of approximately 60–65 years of age. Mean diabetes duration ranged from 7 to 17 years with an HbA1c at baseline of 7.2-8.8%. BMI was slightly higher in the PDM-ProValue population (33.9 kg/m^2^ vs. 28.7–33.6 kg/m^2^). In the PDM-ProValue study all participants were treated with insulin whereas in most CVOTs the number of insulin-treated patients was lower (exception DEVOTE, which compared Insulin degludec to Insulin glargine). The majority of CVOTs included a high proportion of patients with established CVD or at high risk for CVD (> 65%), whilst 89.85% of patients of the PDM-ProValue study had hypertension, 7.7% had atrial fibrillation and 71% had a non-specified diabetes complication. Overall, the population of PDM-ProValue is similar to those of most CVOTs, disregarding the high proportion of participants with established CVD in the latter (Tables 1 and 2). Most CVOTs list glucose-lowering medications introduced after baseline, divided into investigated drug and placebo, shown in absolute numbers and percentages. In the SUSTAIN-6 study, for example, about 20% of participants of both semaglutide groups (0.5 mg/1.0 mg) versus about 40% in the placebo groups adapted their anti-hyperglycaemic regimen during the trial [12]. In 19.5% of participants of the EMPA-REG OUTCOME study treated with empagliflozin, additional glucose-lowering therapy was introduced post-baseline (versus 31.5% in the placebo group [15]). No information about medical reasons, time point or duration of treatment changes are provided, nor are there any details about possible switches from one to another medication. This applies to all CVOTs. In the PDM-ProValue study more participants of the iPDM group received recommendations to adjust their insulin therapy throughout the study than in the control group. Changes in oral antidiabetic medication were negligible in both groups, whereas lifestyle adaptations were more common in the iPDM group [2]. First analysis of the PDM-ProValue study results do not provide information on CV risk and comorbidities of participants of the study.

**Table 1:** Comparison of participants in PDM-ProValue and CVOTs* (all participants)

	PDM-ProValue	CVOTs*
Diabetes type	T2DM	T2DM
Insulin-treated (%)	100	23.5 to 84.2
Male (%)	55.9 to 60.5	61 to 71.2
Age (years), mean	64.5 to 64.9	59.9 to 65.4
Time since diagnosis (years), mean	14.3 to 14.4	7 to 16.6
BMI (kg/m^2^), mean	33.8 to 34.0	28.7 to 33.6
Baseline HbA1c (%)	8.4 to 8.5	7.2 to 8.8
Change in HbA1c (%)	0.2 to 0.5	0.1 to − 1.4

*T2DM* type 2 diabetes mellitus

* ELIXA, LEADER, SUSTAIN6, EXSCEL, Harmony Outcomes, DEVOTE, CANVAS, EMPA-REG, DECLARE-TIMI 58, TECOS, EXAMINE, SAVOR-TIMI & CARMELINA

**Table 2:** Participant characteristics of individual studies (PDM-ProValue and CVOTs [intervention group])

Study	PDM-ProValue (1)	Meta-analysis GLP-1 receptor agonists [29]				Harmony outcomes [14]	Insulin
	iPDM	ELIXA [10]	LEADER [11]	SUSTAIN6 [12]	EXSCEL [13]		DEVOTE [19]
Diabetes type	T2DM	T2DM	T2DM	T2DM	T2DM	T2DM	T2DM
Insulin-treated (%)	100	39	43	58* 58**	46.2	60	84.2
Male (%)	60.5	69	64.5	59.9* 63**	62	70	62.8
Age (years), mean (SD)	64.5 (10.9)	59.9 ± 9.7	64.2 ± 7.2	64.6 ± 7.3* 64.7 ± 7.1**	62.0	64.1 (8.7)	64.9 ± 7.3
Time since diagnosis (years), mean (SD)	14.4 (8.7)	9.2 ± 8.2	12.8 ± 8.0	14.3 ± 8.2* 14.1 ± 8.2**	12.0	14.1 (8.6)	16.6 ± 8.8
Time since start of insulin therapy (years), mean (SD)	7.1 (6.6)	nd	nd	nd	nd	nd	nd
BMI, mean (SD)	33.8 (6.1)	30.1 ± 5.6	32.5 ± 6.3	32.7 (6.29)* 32.9 (6.18)**	31.8	32.3 (5.9)	33.6 ± 6.8
Baseline HbA1c (%), mean (SD)	8.5 (1.1)	7.7 ± 1.3	8.7 ± 1.6	8.7 ± 1.4* 8.7 ± 1.5**	8.0	8.76 (1.5)	8.4 ± 1.6
Change in HbA1c (%)	− 0.5	− 0.6	− 1.1	− 1.1* − 1.4**	− 0.4	− 0.9	− 0.9
Proportion with established CVD (%)	nd	100	82	83	73	70	63.3

Study	SGLT-2 inhibitors			DPP-4 inhibitors			
	CANVAS [16]	EMPA-REG [15]	DECLARE-TIMI 58 [17]	TECOS [8]	EXAMINE [7]	SAVOR-TIMI [6]	CARMELINA [9]
Diabetes type	T2DM	T2DM	T2DM	T2DM	T2DM	T2DM	T2DM
Insulin-treated (%)	49.9	48	41.6	23.5	29.4	41.6	58.8
Male (%)	64.9	71.2	63.1	70.9	67.7	66.6	61.5
Age (years), mean (SD)	63.2 ± 8.3	63.1 ± 8.6	63.9 ± 6.8	65.4 ± 7.9	61.0	65.1 ± 8.5	66.1 (9.1)
Time since diagnosis (years), mean (SD)	13.5 ± 7.7	> 10^#^	11.0	11.6 ± 8.1	7.1	10.3	15.0 (9.6)
Time since start of insulin therapy (years), mean (SD)	nd	nd	nd	nd	nd	nd	nd
BMI, mean (SD)	31.9 ± 5.9	30.6 ± 5.3	32.1 ± 6.0	30.2 ± 5.6	28.7	31.1 ± 5.5	31.4 (5.3)
Baseline HbA1c (%), mean (SD)	8.2	8.07	8.3 ± 1.2	7.2	8.0	8.0	7.9 (1.0)
Change in HbA1c (%)	− 0.3	− 0.26	− 0.4	− 0.1	− 0.33	− 0.3	− 036
Proportion with established CVD (%)	64.8	99.4	41.6	73.6	100	78.4	58.1

*nd* not determined, *T2DM* type 2 diabetes mellitus

* Semaglutide 0.5 mg; ** Semaglutide 1.0 mg; ^#^ In 57% of participants time since diagnosis of type 2 diabetes was > 10 years

### Role of SMBG in PDM-ProValue and CVOTs

To ensure correct interpretation as well as replication of the methodology of CVOTs beyond the dominant diabetes marker HbA1c, reliable glucose information based on SMBG should be part of the study design and protocol. Study results can be affected if SMBG methods and results are addressed inconsistently or not at all.

In most CVOTs—except for EXAMINE, SUSTAIN-6, TECOS, Harmony Outcomes and DELCARE-TIMI 58—the use of SMBG is mentioned in the related study protocol [7, 8, 12, 14, 22]. Only the publication of the DEVOTE study, which investigated insulins, provides further information about recorded values, numbers and nature of resulting therapy changes or other consequences (Table 3 [19]). Generally, SMBG values were solely collected to facilitate anti-diabetic treatment at the investigators discretion but not reported in the results [23]. In the PDM-ProValue study, SMBG was an integral part of the iPDM process. Patients were educated in SMBG utilisation and the study protocol required structured and periodic SMBG and SMBG regimen adaptations if advised by the physician. The systematic documentation and analysis of SMBG data was an additional part of the iPDM process and potential therapy adjustments. Change in SMBG testing frequency was investigated as secondary endpoint in the PDM-ProValue study and even though frequency of SMBG measurement was documented throughout the study, no changes have been reported so far [2].

**Table 3:** Mentioned/reported use of SMBG in CVOTs

	SMBG reported on clinicaltrials.gov	SMBG mentioned in clinical study protocol	SMBG results reported in final publication
*DPP-4 inhibitors*			
SAVOR-TIMI	No (NCT01107886)	Yes	No (6)
EXAMINE	No (NCT00968708)	No	No (7)
TECOS	No (NCT00790205)	No	No (8)
CARMELINA	No (NCT01897532)	Yes	No (9)
*SGLT-2 inhibitors*			
EMPA-REG OUTCOME	No (NCT01131676)	Yes	No (15)
CANVAS	No (NCT01032629)	Yes	No (16)
DECLARE-TIMI 58	No (NCT01730534)	No	No (17)
*GLP-1 receptor agonist*			
ELIXA	No (NCT01147250)	Yes	No (10)
SUSTAIN-6	No (NCT01720446)	No	No (12)
LEADER	No (NCT01179048)	Yes	No (11)
EXCSEL	No (NCT01144338)	Not determined	No (13)
Harmony outcomes	No (NCT02465515)	No	No (14)
*Insulin*			
DEVOTE	No (NCT01959529)	Yes	Yes (19)

In light of the sparse reporting of SMBG measures and according to the Good Clinical Practice standard and the growing evidence for a structured approach of SMBG [24], in addition to a detailed description of SMBG methodology, all relevant findings related to SMBG should be reported in scientific publications of CVOTs. In view of a better comparability of clinical studies, Schnell et al. recommended a structured reporting of SMBG topics in the results section including, for example, the number of SMBG performing patients, evaluation of the performance of the BG systems, pre-/post-prandial and nocturnal glucose levels, details about modulation of diabetes treatment based on SMBG data and between-group differences in SMBG [23].

### Clinical perspectives

**Fig. 2:**
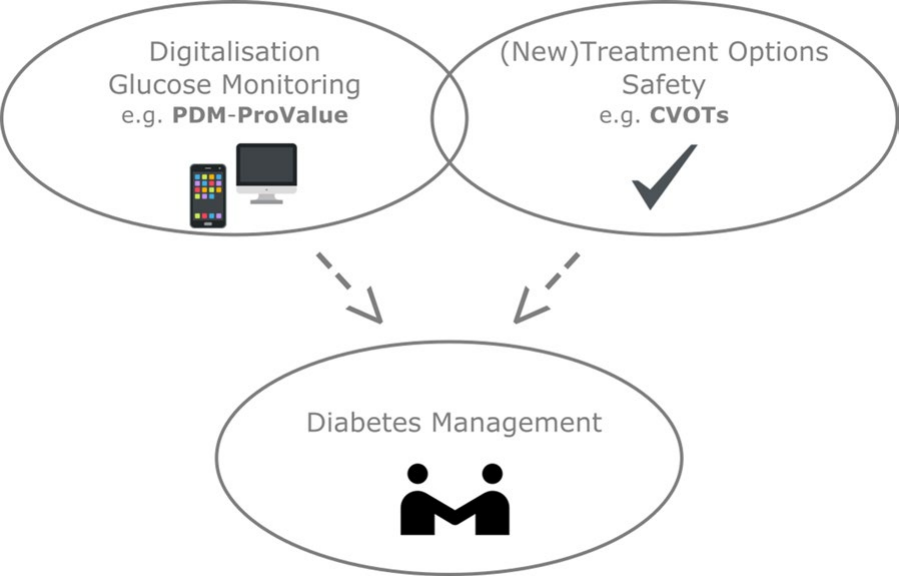
Diabetes management as a combination of new treatment options and improving management tools. CVOTs primarily investigate the safety of (new) treatment options, whereas the PDM-ProValue study is an example of studies analysing the impact of digitalisation and glucose monitoring. Both study approaches included a similar patient population and follow the overall goal to improve diabetes management and consequently the well-being of T2DM patients

Even though CVOTs are primarily safety studies and PDM-ProValue aimed at improving clinical outcomes, both study approaches included a similar patient population and follow the overall goal to improve well-being of T2DM patients by reducing disease-related outcomes (Fig. 2). Results of PDM-ProValue and other studies demonstrate the benefits of digitised diabetes management programs, enabling visualisation, analysis and interpretation of BG data. Nevertheless, there is a discrepancy between rising therapy innovations, digital possibilities and treatment outcomes. Despite pharmacological and technical options, only a minority of patients achieve their treatment goals: 50% of diabetic patients are diagnosed, about 70% are assigned to individual HbA1c target goals by their doctors and about 50% of these patients achieve these goals [25–27]. The main reason for this discrepancy lies in the inadequate realisation of technical possibilities. For example, 78% of all patients using SMBG fill out the measurement protocols by hand and experience has shown that up to 60% of these manual entries are wrong. If the measured data would automatically be transferred to a digital protocol, the error probability would approach zero. On the other hand physicians cannot compete with modern IT: suspicious BG patterns requiring therapy correction were overlooked or misinterpreted by doctors in 20 to 80% of cases even when measurement protocols were already digitally recorded, whilst digital recognition algorithms can reveal nearly all abnormal values in seconds.

With regard to the continuously increasing burden of diabetes worldwide a standardised diabetes management becomes all the more important. Not only could this enhance glycaemic control and thereby reduce comorbidities but it could also provide a tool to optimise treatment options by assessing safety and efficacy of medications the most comparable way possible. If standardised diabetes management became standard of care, comparability of outcome trials would markedly increase, circumventing one of their major limitations. Safety and efficacy could be investigated in an iPDM environment.

Another concept of trials in the field of diabetes are real-world studies. They are generally seen as a good way to complement information provided by randomised controlled trials. Still, discrepancies between randomised controlled trials and real-world data, especially with regard to data quality and integrity of cases impede the direct comparison of results [28]. Standardising diabetes management e.g. by implementing iPDM may thus also strengthen comparability of randomised controlled trials and real-world studies.

## Conclusion

Both, PDM-ProValue and CVOTs are landmark study areas in the field of diabetology. Results of CVOTs have already found their way into guidelines and so should the ever increasing importance of standardised and also digitalised diabetes management as presented exemplarily by the PDM-ProValue study. The consideration of both areas of study in the clinical and practical setting will further enhance the optimisation of personalised diabetes management.

## Abbreviations

BG: blood glucose
CV: cardiovascular
CVD: cardiovascular disease
CVOT: cardiovascular outcome trials
DPP-4: dipeptidyl peptidase-4
GLP-1: glucagon-like peptide-1
iPDM: integrated personalised diabetes management
MACE: major adverse cardiac event
SGLT-2: sodium–glucose cotransporter-2
SMBG: self-monitoring of blood glucose
T2DM: type 2 diabetes mellitus
